# Crystal structure and Hirshfeld surface analysis of pulcherrin J

**DOI:** 10.1107/S2056989017011239

**Published:** 2017-09-29

**Authors:** K. Osahon Ogbeide, Mujeeb-Ur-Rehman, J. Bodunde Owolabi, Abiodun Falodun, M. Iqbal Choudhary, Sammer Yousuf

**Affiliations:** a Department of Chemistry, Faculty of Physical Sciences, University of Benin, Benin City, Nigeria; b H.E.J. Research Institute of Chemistry, International Center for Chemical and Biological Sciences, University of Karachi, Karachi 75270, Pakistan; c Department of Chemistry, School of Sciences, The Federal University of Technology, Akure, Nigeria; d Department of Pharmaceutical Chemistry, Faculty of Pharmacy, University of Benin, Benin City, Nigeria

**Keywords:** crystal structure, Pulcherrin J, *Caesalpinia pulcherrima* (*L*.), diterpene, Hirshfeld surface analysis

## Abstract

The title compound, C_29_H_36_O_4_ [systematic name (4a*R*,5*R*,6a*S*,7*R*,11a*S*,11b*R*)-4a-hydroxy-4,4,7,11b-tetramethyl-1,2,3,4,4a,5,6,6a,7,11,11a,11b-dodecahydrophen­anthro[3,2-*b*]furan-5-yl cinnamate], a natural diterpene known as pulcherrin J, was isolated from stem barks of medicinally important *Caesalpinia pulcherrima* (*L*.). The crystal structure of pulcherrin J shows it to be composed of a central core of three *trans*-fused cyclo­hexane rings and a near planar five-membered furan ring, along with an axially oriented cinnamate moiety and an ­hydroxy substituent attached at positions 4a and 5 of the steroid ring system, respectively. The absolute structure was established with the use of Cu *K*α radiation. In the crystal, mol­ecules are linked by O—H⋯O hydrogen bonds to generate [100] *C*(8) chains. Hirshfeld surface analysis indicates that the most significant contacts in packing are H⋯H (67.5%), followed by C⋯H (19.6%) and H⋯O (12.9%).

## Abstract 2

The natural diterpene known as pulcherrin J was isolated from stem barks of medicinally important *Caesalpinia pulcherrima* (*L*.). The mol­ecule is composed of a central core of three *trans*-fused cyclo­hexane rings and a near planar five-membered furan ring, along with an axially oriented cinnamate moiety and a­hydroxyl substituent attached to the C-8 and C-9 positions of the steroid ring system, respectively. Hirshfeld surface analysis indicates that the most significant contacts in packing are H⋯H (67.5%), followed by C⋯H (19.6%) and H⋯O (12.9%).

## Chemical context   

*Caesalpinia pulcherrima* (L.) is one of the widely cultivated species of the genus *Caesalpinia*. It is an ornamental plant with attractive inflorescence in yellow, red, and orange, generally blooming in winter. Its small size and tolerability towards pruning allows it to be grown in groups to form a windbreak. It can also be used to create a center of attention for humming birds (Frisch *et al.*, 2005 ▸). In addition to the ornamental value, *C. pulcherrima* has been known to exhibit cytotoxic (Promsawan *et al.*, 2003 ▸; McPherson *et al.*, 1986 ▸), anti­tubercular (Promsawan *et al.*, 2003 ▸), anti­bacterial, anti­fungal (Ragasa *et al.*, 2002 ▸), and leishmanicidal (Erharuyi *et al.*, 2016 ▸) activities. The compounds isolated from *C. pulcherrima* are also reported to be active against DNA repair-deficient yeast mutant (Patil *et al.*, 1997 ▸). The plants of genus *Caesalpinia*, including *C. pulcherrima*, are known to be a rich source of cassane-type diterpenoids. The literature reports the isolation of a number of cassane-type diterpenoids from the stems, and root barks, such as pulcherrimins A–F, and pulcherrins A–R (Erharuyi *et al.*, 2017 ▸; Yodsaoue *et al.*, 2011 ▸; Pranithanchai *et al.*, 2009 ▸; Roach *et al.*, 2003 ▸). In continuation of our work on the phytochemical investigation of medicinally important plants, we have isolated the crystalline pulcherrin J, a cassane-type diterpenoid, previously reported by Erharuyi and co-workers (Erharuyi *et al.*, 2017 ▸). To the best of our knowledge, this is the first report of the the crystal structure and the Hirshfeld surface analysis of pulcherrin J.
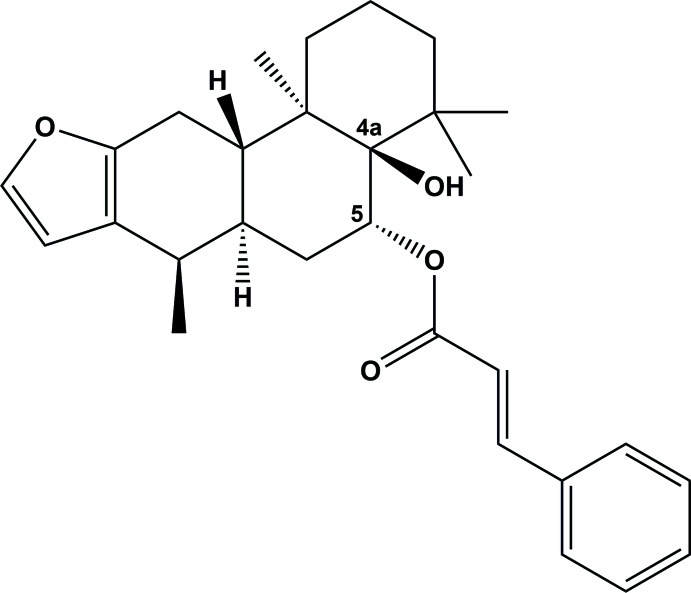


## Structural commentary   

**Figure 1:**
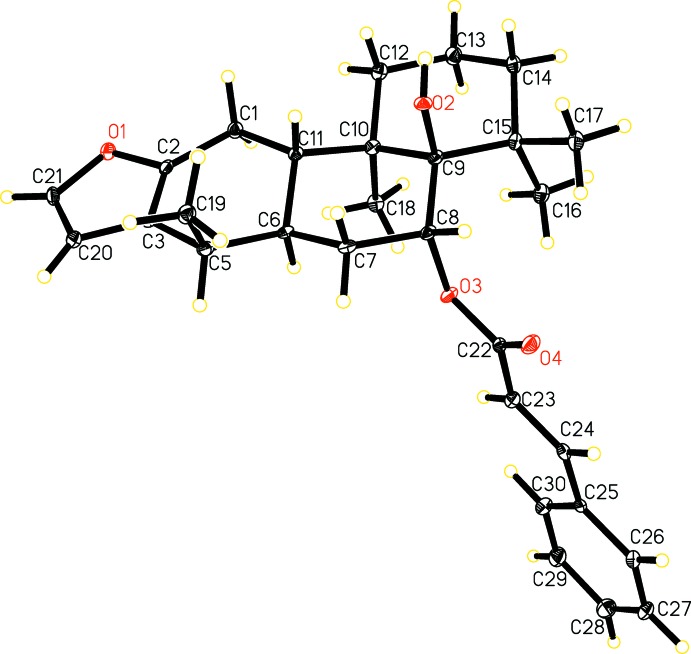
The mol­ecular structure, with displacement ellipsoids drawn at the 30% probability level.

The title compound (Fig. 1 ▸) is a cassane-type diterpenoid comprising of three cyclo­hexane rings *A* (C1–C3/C5–C7), *B* (C6–C11) and *C* (C9–10/C12–C15) and an almost planar five-membered furan ring (O1/C2–C3/C20–C21) fused to ring *A* along the C2—C3 bond. Cyclo­hexane rings *A*, *B*, and *C* are *trans* fused to each other along the C6—C11 and C8—C9 bonds and attain half-chair, chair and chair conformations, respectively, as observed in related structures (Gómez-Hurtado *et al.*, 2013 ▸; Fun *et al.*, 2010*a*, ▸ *b* ▸; Matsuno *et al.*, 2008 ▸; Ruggiero *et al.*, 1997 ▸). The *axially* oriented cinnamate group (O3/O4/C22–C30) and hydroxy moieties at C8 and C9 of ring *B*, respectively, are trans to each other [O2—C9—C8—O3 = −171.41 (13)°]. The dihedral angle between the furan and phenyl ring of the cinnamate moiety is 83.77 (16)°. The absolute configurations of the stereogenic centres are C5 *R*, C6 *R*, C8 *R*, C9 *R*, C10 *R* and C11 *S*. The conformation of the mol­ecule is consolidated by a C1*8*—H18*A*⋯O3 intra­molecular inter­action, forming an *S*(6) graph-set ring motif.

## Hydrogen bonding and Hirshfeld surface analysis   

**Figure 2:**
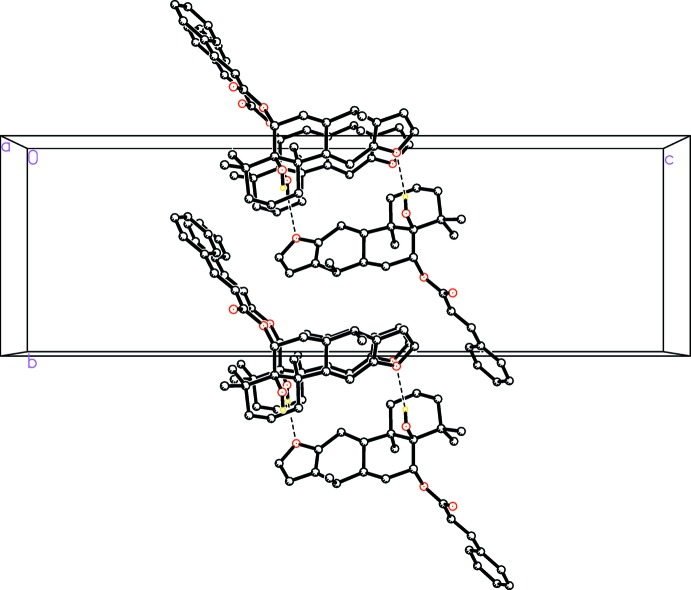
The crystal packing. H atoms involved in hydrogen bonding are shown.

**Figure 3:**
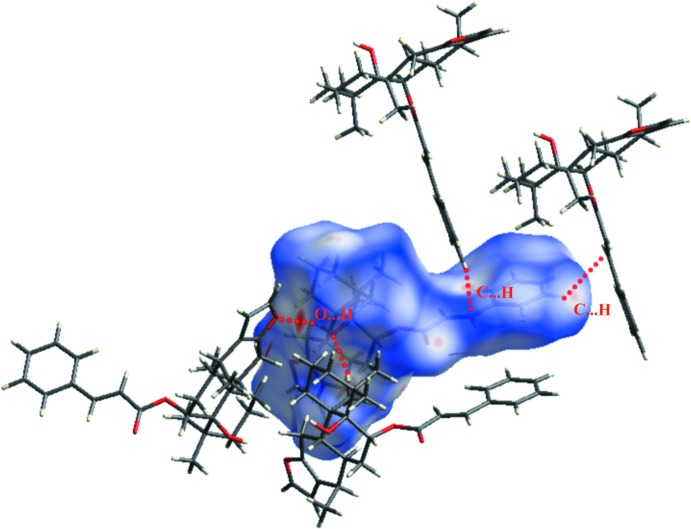
*d*_norm_ mapped on the Hirshfeld surface for visualizing the contacts of the title compound. Dotted lines indicate hydrogen bonds.

**Figure 4:**
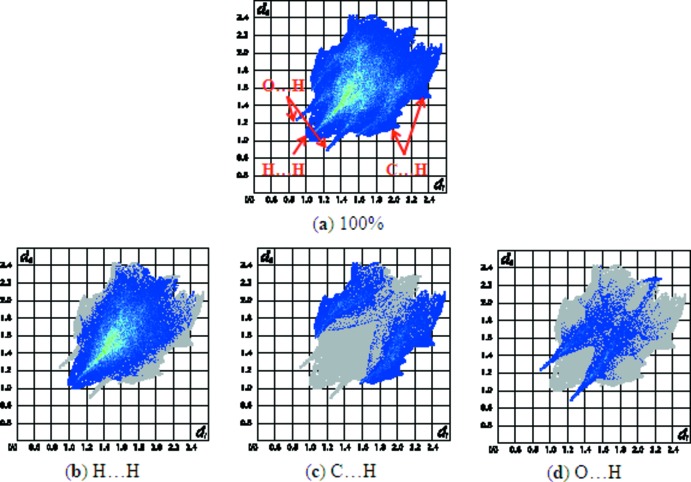
(*a*) Fingerprint plot of the title compound, (*b*–*d*) H⋯H, C⋯H and O⋯H contacts. The outline of the full fingerprint plots is shown in grey. *d*_i_ is the closet inter­nal distance from a given point on the Hirshfeld surface and *d*_e_ is the closest external contact.

In the crystal, the mol­ecules are connected by O2—H2*A*⋯O1^i^ inter­actions to generate *C*(8) chains propagating in the [100] direction. (Table 1 ▸, Fig. 2 ▸). The Hirshfeld surface analysis (Spackman *et al.*, 2009 ▸) indicates that the percentage contribution of H⋯H inter­actions to the packing is 67.5% (Fig. 3 ▸). Other important inter­actions based upon the percentages are C⋯H (19.6%) and H⋯O (12.9%), as shown in the fingerprint plots, in which cyan dots indicate the percentage of the inter­action over the total Hirshfeld surface (Fig. 4 ▸).

## Comparison with reported literature   

Structurally the title compound is similar to the reported isovouacapenol C (Fun *et al.*, 2010*b* ▸) with the difference that no hy­droxy substituent occurs on ring *B*, while the benzoate moiety is replaced by a cinnamate moiety. The O—H⋯O hydrogen bond is the most important contributor to the crystal packing of pulcherrin J, and other related structures such as isovouacapenol C and vouacapen-5a-ol (Fun *et al.*, 2010*a*, ▸ *b* ▸), all of which lead to chains in the crystal.

## Isolation and crystallization   

2.5 kg of ground *C. pulcherrima* (*L*.) Swartz stem bark was soaked in methanol (7.5 l) at ambient temperature: 220 g of crude extract was obtained after filtration and concentration, by using a rotary evaporator at 318 K. 200 g of the crude extract was fractionated by using silica gel chromatography, first with hexane (9.4 l) followed by increasing polarities with *n*-hexa­ne:ethyl­acetate (1:1) (12.5 l), ethyl acetate (8.2 l), ethyl acetate:methanol (1:1) (13 l) and methanol (7 l). Concentration of the different fractions *in vacuo* gave five different fractions of 0.45 g (0.23%), 38.81 g (19.41%), 25.75 g (12.75%), 127.73 g (63.87%) and 4.18 g (2.09%) obtained on elution with *n*-hexane, *n*-hexa­ne:ethyl­acetate (1:1), ethyl acetate, ethyl acetate:methanol (1:1) and methanol, respectively. The fraction obtained on elution with *n*-hexa­ne:ethyl acetate­(1:1) was re-chromatographed over silica gel (SiO_2_, 6.5 × 135 cm column) by using increasing proportions of *n*-hexane with ethyl acetate [100:0 (7.5 l), 95:5 (10 l), 90:10 (24.5 l), 85:15 (7.5 l), 80:20 (6 l), and 0:100 (4.5 l)]. Each obtained fraction (250 ml of each) was monitored carefully on TLC and combined into 12 main fractions named as CP4–9, CP10–17, CP18–33, CP34–48, CP49–61, CP63–76, CP77–92, CP93–123,CP124–135, CP136–139, CP140–145 and CP153–162. The fraction obtained on elution with *n*-hexa­ne:ethyl acetate 95:5 gave crystalline precipitates, which were suspended in *n*-hexane, filtered and dried to obtain purified crystalline pulcherrin J (130.4 mg).

^1^H NMR (400MHz C_3_D_6_O): 8.08 (*bd*, *J* = 7.2 H31,71), 7.64 (*bt*, *J* = 7.6, H51), 7.53 (*bt*, *J* = 7.2, Hz H41,61), 7.27 (*d*, *J* = 1.6Hz, H16), 6.20 (*d*, *J* = 2, H15), 5.6 (*t*, *J* = 3.0, H6), 2.62–2.51 (*m*, H9), 2.58 (*m*, H14), 2.46 (*m*, H11), 2.41–2.33 (*m*, H7*b*); 1.59–1.52 (*m* H7*a*), 2.13–2.07 (*m*, H8), 1.56 (*s*, H20), 1.21 (*s*, H19), 1.03 (*s*, H18), 0.98 (*d*, *J* = 6.8 Hz, H17), 1.98–1.89 (*m*, H3*b*); 1.05 (*m*, H3*a*), 1.79–1.77 (*m*, H2*b*); 1.49–1.47 (*m*, H2*a*), 1.76–1.74 (*m*, H1*b*); 1.45–1.43 (*m*, H1*a*) ppm. ^13^C NMR (400 MHz C_3_D_6_O) 165.8, 133.1, 129.7, 128.6, 149.5, 140.4, 122.4, 109.5, 76.4, 72.8, 41.3, 39.0, 38.1, 38.0, 34.9, 31.6, 31.2, 30.7, 27.8, 26.0, 21.9, 18.3, 17.6, 17.2 ppm. IR (CH_3_OH, cm^−1^): 3593.0, 2929.6, 2869.1, 1705.9, 1635.8, 1505.4, 1458.4, 1392.3, 1316.2, 1283.0, 1176.9, 1007.6, 929.7, 733.0.

## Refinement   

Crystal data, data collection and structure refinement details are summarized in Table 2 ▸. H atoms were located in a difference-Fourier map, positioned with idealized geometry and refined with *U*_iso_(H) = 1.5*U*_eq_, C—H = 0.97 Å for CH_3_, 1.2*U*_eq_, C—H = 0.97 Å for CH_2_ and C—H = 0.93 Å for olefinic and aromatic CH. The hydrogen atom on the oxygen [O—H= 0.82 (3) Å] was located in difference-Fourier map and refined isotropically.

## Supplementary Material

Crystal structure: contains datablock(s) global, I. DOI: 10.1107/S2056989017011239/hb7687sup1.cif

Structure factors: contains datablock(s) I. DOI: 10.1107/S2056989017011239/hb7687Isup2.hkl

Supporting information file. DOI: 10.1107/S2056989017011239/hb7687Isup3.cml

CCDC reference: 1565682

Additional supporting information:  crystallographic information; 3D view; checkCIF report

## Figures and Tables

**Table 1:** Hydrogen-bond geometry (Å, °)

*D*—H⋯*A*	*D*—H	H⋯*A*	*D*⋯*A*	*D*—H⋯*A*
O2—H2*A*⋯O1^i^	0.82 (3)	2.28 (3)	3.067 (2)	160 (2)
C18—H18*A*⋯O3	0.98	2.23	3.039 (2)	139

Symmetry code: (i) 
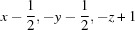
.

**Table 2:** Experimental details

Crystal data	
Chemical formula	C_29_H_36_O_4_
*M*_r_	448.58
Crystal system, space group	Orthorhombic, *P*2_1_2_1_2_1_
Temperature (K)	100
*a*, *b*, *c* (Å)	6.6663 (3), 10.6256 (5), 33.3005 (17)
*V* (Å^3^)	2358.8 (2)
*Z*	4
Radiation type	Cu *K*α
μ (mm^−1^)	0.65
Crystal size (mm)	0.13 × 0.12 × 0.08

Data collection	
Diffractometer	Bruker SMART APEX CCD
Absorption correction	Multi-scan (*SADABS*; Bruker, 2009 ▸)
*T*_min_, *T*_max_	0.920, 0.950
No. of measured, independent and observed [*I* > 2σ(*I*)] reflections	18537, 4124, 3499
*R*_int_	0.074
(sin θ/λ)_max_ (Å^−1^)	0.596

Refinement	
*R*[*F* ^2^ > 2σ(*F* ^2^)], *wR*(*F* ^2^), *S*	0.038, 0.082, 1.05
No. of reflections	4124
No. of parameters	306
H-atom treatment	H atoms treated by a mixture of independent and constrained refinement
Δρ_max_, Δρ_min_ (e Å^−3^)	0.15, −0.27
Absolute structure	Flack (1983 ▸)
Absolute structure parameter	−0.18 (19)

Computer programs: *SMART* and *SAINT* (Bruker, 2009 ▸), *SHELXT2014* (Sheldrick, 2015*a* ▸), *SHELXL2016* (Sheldrick, 2015*b* ▸), *SHELXTL* (Sheldrick, 2008 ▸), *PLATON* (Spek, 2009 ▸) and *publCIF* (Westrip, 2010 ▸).

## supplementary crystallographic information

### Crystal data

**Table:**

C_29_H_36_O_4_	*D*_x_ = 1.263 Mg m^−^^3^
*M**_r_* = 448.58	Cu *K*α radiation, λ = 1.54178 Å
Orthorhombic, *P*2_1_2_1_2_1_	Cell parameters from 6933 reflections
*a* = 6.6663 (3) Å	θ = 2.7–66.5°
*b* = 10.6256 (5) Å	µ = 0.65 mm^−^^1^
*c* = 33.3005 (17) Å	*T* = 100 K
*V* = 2358.8 (2) Å^3^	Plate, colourless
*Z* = 4	0.13 × 0.12 × 0.08 mm
*F*(000) = 968	

### Data collection

**Table:**

Bruker SMART APEX CCD diffractometer	4124 independent reflections
Radiation source: fine-focus sealed tube	3499 reflections with *I* > 2σ(*I*)
Graphite monochromator	*R*_int_ = 0.074
ω scans	θ_max_ = 66.8°, θ_min_ = 2.7°
Absorption correction: multi-scan (SADABS; Bruker, 2009)	*h* = −7→4
*T*_min_ = 0.920, *T*_max_ = 0.950	*k* = −11→12
18537 measured reflections	*l* = −39→39

### Refinement

**Table:**

Refinement on *F*^2^	Secondary atom site location: difference Fourier map
Least-squares matrix: full	Hydrogen site location: inferred from neighbouring sites
*R*[*F*^2^ > 2σ(*F*^2^)] = 0.038	H atoms treated by a mixture of independent and constrained refinement
*wR*(*F*^2^) = 0.082	*w* = 1/[σ^2^(*F*_o_^2^) + (0.0398*P*)^2^ + 0.0469*P*] where *P* = (*F*_o_^2^ + 2*F*_c_^2^)/3
*S* = 1.05	(Δ/σ)_max_ < 0.001
4124 reflections	Δρ_max_ = 0.15 e Å^−^^3^
306 parameters	Δρ_min_ = −0.27 e Å^−^^3^
0 restraints	Absolute structure: Flack (1983)
Primary atom site location: structure-invariant direct methods	Absolute structure parameter: −0.18 (19)

### Special details

**Table:**

Geometry. All esds (except the esd in the dihedral angle between two l.s. planes) are estimated using the full covariance matrix. The cell esds are taken into account individually in the estimation of esds in distances, angles and torsion angles; correlations between esds in cell parameters are only used when they are defined by crystal symmetry. An approximate (isotropic) treatment of cell esds is used for estimating esds involving l.s. planes.
Refinement. Refinement of F^2^ against ALL reflections. The weighted R-factor wR and goodness of fit S are based on F^2^, conventional R-factors R are based on F, with F set to zero for negative F^2^. The threshold expression of F^2^ > 2sigma(F^2^) is used only for calculating R-factors(gt) etc. and is not relevant to the choice of reflections for refinement. R-factors based on F^2^ are statistically about twice as large as those based on F, and R- factors based on ALL data will be even larger.

### Fractional atomic coordinates and isotropic or equivalent isotropic displacement parameters (Å^2^)

**Table:**

	*x*	*y*	*z*	*U*_iso_*/*U*_eq_	
O1	0.37182 (19)	−0.05535 (13)	0.57637 (4)	0.0171 (3)	
O2	−0.0950 (2)	−0.16046 (14)	0.40894 (4)	0.0144 (3)	
O3	0.12570 (19)	0.13663 (12)	0.38017 (4)	0.0154 (3)	
O4	−0.1179 (2)	0.21242 (13)	0.33934 (4)	0.0201 (3)	
C1	0.2954 (3)	−0.11384 (19)	0.50647 (5)	0.0154 (4)	
H1A	0.2679	−0.2023	0.5140	0.018*	
H1B	0.4367	−0.1082	0.4974	0.018*	
C2	0.2654 (3)	−0.03178 (19)	0.54176 (6)	0.0138 (4)	
C3	0.1496 (3)	0.07066 (18)	0.54590 (6)	0.0140 (4)	
C5	0.0101 (3)	0.1140 (2)	0.51340 (5)	0.0146 (4)	
H5	0.0140	0.2081	0.5128	0.017*	
C6	0.0901 (3)	0.06556 (18)	0.47230 (6)	0.0124 (4)	
H6	0.2106	0.1170	0.4652	0.015*	
C7	−0.0671 (3)	0.08691 (18)	0.43950 (6)	0.0142 (4)	
H7A	−0.1916	0.0427	0.4475	0.017*	
H7B	−0.0980	0.1780	0.4385	0.017*	
C8	−0.0108 (3)	0.04400 (18)	0.39731 (6)	0.0131 (4)	
H8	−0.1356	0.0430	0.3807	0.016*	
C9	0.0806 (3)	−0.08998 (18)	0.39668 (5)	0.0130 (4)	
C10	0.2459 (3)	−0.10865 (19)	0.42973 (6)	0.0134 (4)	
C11	0.1536 (3)	−0.07380 (18)	0.47164 (5)	0.0118 (4)	
H11	0.0282	−0.1246	0.4746	0.014*	
C12	0.3090 (3)	−0.24846 (18)	0.43029 (6)	0.0155 (4)	
H12A	0.4232	−0.2586	0.4490	0.019*	
H12B	0.1960	−0.2993	0.4408	0.019*	
C13	0.3700 (3)	−0.3000 (2)	0.38903 (6)	0.0198 (5)	
H13A	0.4947	−0.2579	0.3801	0.024*	
H13B	0.3975	−0.3912	0.3913	0.024*	
C14	0.2051 (3)	−0.27836 (19)	0.35786 (6)	0.0188 (5)	
H14A	0.0863	−0.3291	0.3654	0.023*	
H14B	0.2530	−0.3090	0.3315	0.023*	
C15	0.1407 (3)	−0.13918 (19)	0.35356 (6)	0.0167 (5)	
C16	0.3124 (3)	−0.0690 (2)	0.33183 (6)	0.0242 (5)	
H16A	0.2714	0.0179	0.3264	0.036*	
H16B	0.3425	−0.1115	0.3064	0.036*	
H16C	0.4323	−0.0690	0.3489	0.036*	
C17	−0.0416 (3)	−0.1347 (2)	0.32547 (6)	0.0231 (5)	
H17A	−0.1533	−0.1808	0.3377	0.035*	
H17B	−0.0068	−0.1734	0.2997	0.035*	
H17C	−0.0812	−0.0469	0.3211	0.035*	
C18	0.4348 (3)	−0.02606 (19)	0.42266 (6)	0.0161 (4)	
H18A	0.3971	0.0497	0.4077	0.024*	
H18B	0.5341	−0.0740	0.4072	0.024*	
H18C	0.4925	−0.0018	0.4486	0.024*	
C19	−0.2055 (3)	0.0740 (2)	0.52362 (6)	0.0175 (4)	
H19A	−0.2174	−0.0176	0.5213	0.026*	
H19B	−0.2990	0.1144	0.5049	0.026*	
H19C	−0.2375	0.0998	0.5511	0.026*	
C20	0.1840 (3)	0.11595 (19)	0.58590 (6)	0.0172 (4)	
H20	0.1234	0.1873	0.5981	0.021*	
C21	0.3183 (3)	0.03823 (19)	0.60280 (6)	0.0187 (5)	
H21	0.3693	0.0467	0.6293	0.022*	
C22	0.0525 (3)	0.21390 (18)	0.35149 (6)	0.0152 (4)	
C23	0.2113 (3)	0.29919 (19)	0.33754 (6)	0.0169 (4)	
H23	0.3381	0.2981	0.3506	0.020*	
C24	0.1815 (3)	0.37822 (18)	0.30697 (6)	0.0167 (4)	
H24	0.0526	0.3762	0.2948	0.020*	
C25	0.3260 (3)	0.46782 (18)	0.29013 (5)	0.0161 (5)	
C26	0.2651 (3)	0.5491 (2)	0.25947 (6)	0.0215 (5)	
H26	0.1334	0.5421	0.2488	0.026*	
C27	0.3945 (4)	0.6398 (2)	0.24439 (6)	0.0266 (5)	
H27	0.3517	0.6940	0.2234	0.032*	
C28	0.5861 (4)	0.6511 (2)	0.26003 (6)	0.0280 (5)	
H28	0.6742	0.7145	0.2503	0.034*	
C29	0.6485 (3)	0.5695 (2)	0.29001 (6)	0.0251 (5)	
H29	0.7807	0.5765	0.3004	0.030*	
C30	0.5218 (3)	0.4784 (2)	0.30487 (6)	0.0216 (5)	
H30	0.5675	0.4225	0.3252	0.026*	
H2A	−0.075 (4)	−0.237 (3)	0.4105 (7)	0.036 (8)*	

### Atomic displacement parameters (Å^2^)

**Table:**

	*U*^11^	*U*^22^	*U*^33^	*U*^12^	*U*^13^	*U*^23^
O1	0.0206 (7)	0.0181 (8)	0.0127 (7)	−0.0011 (6)	−0.0039 (6)	0.0009 (6)
O2	0.0138 (7)	0.0121 (8)	0.0174 (7)	−0.0050 (6)	−0.0006 (5)	0.0018 (6)
O3	0.0167 (7)	0.0145 (8)	0.0151 (7)	−0.0016 (6)	−0.0021 (6)	0.0062 (6)
O4	0.0215 (8)	0.0202 (8)	0.0187 (7)	−0.0006 (6)	−0.0053 (6)	0.0057 (6)
C1	0.0169 (10)	0.0159 (11)	0.0134 (10)	0.0030 (9)	−0.0012 (8)	0.0008 (8)
C2	0.0160 (11)	0.0154 (12)	0.0101 (9)	−0.0050 (9)	−0.0021 (8)	0.0034 (8)
C3	0.0175 (11)	0.0116 (11)	0.0128 (9)	−0.0043 (8)	0.0022 (8)	0.0029 (8)
C5	0.0167 (10)	0.0119 (11)	0.0151 (10)	0.0000 (8)	0.0017 (8)	0.0017 (9)
C6	0.0124 (10)	0.0103 (10)	0.0145 (9)	−0.0023 (9)	0.0015 (8)	0.0042 (8)
C7	0.0151 (11)	0.0104 (11)	0.0171 (10)	−0.0006 (9)	0.0006 (8)	0.0034 (8)
C8	0.0129 (10)	0.0130 (11)	0.0134 (10)	−0.0041 (8)	−0.0018 (8)	0.0040 (9)
C9	0.0132 (10)	0.0133 (11)	0.0123 (10)	−0.0017 (8)	0.0007 (8)	0.0027 (8)
C10	0.0115 (10)	0.0143 (12)	0.0143 (10)	0.0000 (8)	−0.0003 (8)	0.0014 (8)
C11	0.0101 (9)	0.0129 (10)	0.0125 (9)	−0.0023 (8)	−0.0008 (7)	0.0020 (8)
C12	0.0130 (10)	0.0180 (12)	0.0154 (10)	0.0038 (9)	−0.0021 (8)	0.0020 (9)
C13	0.0239 (12)	0.0191 (12)	0.0164 (10)	0.0053 (9)	0.0028 (8)	−0.0009 (9)
C14	0.0251 (11)	0.0189 (12)	0.0124 (10)	0.0011 (10)	0.0033 (9)	−0.0027 (9)
C15	0.0234 (12)	0.0167 (12)	0.0101 (9)	−0.0004 (9)	0.0001 (8)	0.0004 (9)
C16	0.0350 (13)	0.0240 (13)	0.0135 (10)	−0.0009 (11)	0.0067 (9)	0.0002 (9)
C17	0.0342 (13)	0.0211 (13)	0.0140 (10)	0.0024 (10)	−0.0052 (9)	−0.0005 (10)
C18	0.0118 (10)	0.0200 (12)	0.0166 (10)	−0.0004 (8)	−0.0002 (8)	−0.0011 (9)
C19	0.0179 (10)	0.0187 (12)	0.0159 (10)	0.0020 (9)	0.0034 (9)	0.0011 (9)
C20	0.0214 (11)	0.0167 (11)	0.0134 (9)	−0.0028 (9)	0.0035 (8)	0.0005 (8)
C21	0.0252 (12)	0.0211 (12)	0.0097 (9)	−0.0069 (10)	0.0004 (9)	−0.0026 (9)
C22	0.0240 (12)	0.0118 (11)	0.0097 (9)	0.0041 (9)	−0.0005 (8)	−0.0006 (8)
C23	0.0213 (11)	0.0158 (11)	0.0136 (9)	−0.0021 (9)	−0.0010 (8)	0.0017 (9)
C24	0.0235 (11)	0.0153 (11)	0.0113 (9)	0.0006 (9)	0.0008 (9)	−0.0038 (8)
C25	0.0294 (12)	0.0101 (11)	0.0086 (9)	0.0003 (9)	0.0020 (9)	−0.0012 (8)
C26	0.0337 (13)	0.0183 (13)	0.0126 (10)	0.0008 (10)	−0.0040 (9)	−0.0009 (9)
C27	0.0472 (15)	0.0196 (12)	0.0132 (10)	−0.0034 (12)	−0.0014 (10)	0.0054 (9)
C28	0.0395 (14)	0.0236 (13)	0.0208 (11)	−0.0101 (11)	0.0070 (10)	0.0051 (10)
C29	0.0314 (13)	0.0258 (13)	0.0181 (11)	−0.0052 (10)	0.0017 (9)	0.0025 (10)
C30	0.0296 (13)	0.0190 (12)	0.0163 (10)	−0.0002 (9)	0.0000 (10)	0.0037 (9)

### Geometric parameters (Å, º)

**Table:**

O1—C21	1.375 (2)	C14—C15	1.547 (3)
O1—C2	1.376 (2)	C14—H14A	0.9900
O2—C9	1.448 (2)	C14—H14B	0.9900
O2—H2A	0.82 (3)	C15—C17	1.534 (3)
O3—C22	1.351 (2)	C15—C16	1.546 (3)
O3—C8	1.457 (2)	C16—H16A	0.9800
O4—C22	1.206 (2)	C16—H16B	0.9800
C1—C2	1.477 (3)	C16—H16C	0.9800
C1—C11	1.556 (3)	C17—H17A	0.9800
C1—H1A	0.9900	C17—H17B	0.9800
C1—H1B	0.9900	C17—H17C	0.9800
C2—C3	1.342 (3)	C18—H18A	0.9800
C3—C20	1.435 (3)	C18—H18B	0.9800
C3—C5	1.499 (3)	C18—H18C	0.9800
C5—C19	1.537 (3)	C19—H19A	0.9800
C5—C6	1.556 (3)	C19—H19B	0.9800
C5—H5	1.0000	C19—H19C	0.9800
C6—C7	1.530 (3)	C20—C21	1.342 (3)
C6—C11	1.540 (3)	C20—H20	0.9500
C6—H6	1.0000	C21—H21	0.9500
C7—C8	1.524 (3)	C22—C23	1.469 (3)
C7—H7A	0.9900	C23—C24	1.335 (3)
C7—H7B	0.9900	C23—H23	0.9500
C8—C9	1.549 (3)	C24—C25	1.466 (3)
C8—H8	1.0000	C24—H24	0.9500
C9—C10	1.570 (3)	C25—C26	1.397 (3)
C9—C15	1.580 (3)	C25—C30	1.399 (3)
C10—C12	1.544 (3)	C26—C27	1.388 (3)
C10—C18	1.553 (3)	C26—H26	0.9500
C10—C11	1.570 (3)	C27—C28	1.385 (3)
C11—H11	1.0000	C27—H27	0.9500
C12—C13	1.534 (3)	C28—C29	1.387 (3)
C12—H12A	0.9900	C28—H28	0.9500
C12—H12B	0.9900	C29—C30	1.377 (3)
C13—C14	1.529 (3)	C29—H29	0.9500
C13—H13A	0.9900	C30—H30	0.9500
C13—H13B	0.9900		
			
C21—O1—C2	105.71 (15)	C13—C14—C15	113.94 (16)
C9—O2—H2A	113.2 (18)	C13—C14—H14A	108.8
C22—O3—C8	117.51 (15)	C15—C14—H14A	108.8
C2—C1—C11	110.44 (16)	C13—C14—H14B	108.8
C2—C1—H1A	109.6	C15—C14—H14B	108.8
C11—C1—H1A	109.6	H14A—C14—H14B	107.7
C2—C1—H1B	109.6	C17—C15—C16	106.63 (16)
C11—C1—H1B	109.6	C17—C15—C14	107.83 (16)
H1A—C1—H1B	108.1	C16—C15—C14	107.38 (16)
C3—C2—O1	111.00 (17)	C17—C15—C9	110.07 (16)
C3—C2—C1	129.70 (18)	C16—C15—C9	116.97 (17)
O1—C2—C1	119.26 (17)	C14—C15—C9	107.63 (15)
C2—C3—C20	105.98 (17)	C15—C16—H16A	109.5
C2—C3—C5	122.13 (17)	C15—C16—H16B	109.5
C20—C3—C5	131.80 (18)	H16A—C16—H16B	109.5
C3—C5—C19	109.58 (15)	C15—C16—H16C	109.5
C3—C5—C6	108.72 (15)	H16A—C16—H16C	109.5
C19—C5—C6	115.06 (16)	H16B—C16—H16C	109.5
C3—C5—H5	107.7	C15—C17—H17A	109.5
C19—C5—H5	107.7	C15—C17—H17B	109.5
C6—C5—H5	107.7	H17A—C17—H17B	109.5
C7—C6—C11	108.71 (15)	C15—C17—H17C	109.5
C7—C6—C5	110.13 (15)	H17A—C17—H17C	109.5
C11—C6—C5	115.12 (15)	H17B—C17—H17C	109.5
C7—C6—H6	107.5	C10—C18—H18A	109.5
C11—C6—H6	107.5	C10—C18—H18B	109.5
C5—C6—H6	107.5	H18A—C18—H18B	109.5
C8—C7—C6	116.43 (16)	C10—C18—H18C	109.5
C8—C7—H7A	108.2	H18A—C18—H18C	109.5
C6—C7—H7A	108.2	H18B—C18—H18C	109.5
C8—C7—H7B	108.2	C5—C19—H19A	109.5
C6—C7—H7B	108.2	C5—C19—H19B	109.5
H7A—C7—H7B	107.3	H19A—C19—H19B	109.5
O3—C8—C7	108.23 (15)	C5—C19—H19C	109.5
O3—C8—C9	111.69 (15)	H19A—C19—H19C	109.5
C7—C8—C9	112.61 (15)	H19B—C19—H19C	109.5
O3—C8—H8	108.1	C21—C20—C3	106.84 (18)
C7—C8—H8	108.1	C21—C20—H20	126.6
C9—C8—H8	108.1	C3—C20—H20	126.6
O2—C9—C8	98.81 (14)	C20—C21—O1	110.47 (16)
O2—C9—C10	107.73 (14)	C20—C21—H21	124.8
C8—C9—C10	112.52 (15)	O1—C21—H21	124.8
O2—C9—C15	106.84 (15)	O4—C22—O3	124.71 (18)
C8—C9—C15	114.60 (15)	O4—C22—C23	125.53 (18)
C10—C9—C15	114.68 (15)	O3—C22—C23	109.76 (16)
C12—C10—C18	108.96 (15)	C24—C23—C22	121.45 (18)
C12—C10—C11	108.84 (15)	C24—C23—H23	119.3
C18—C10—C11	108.63 (15)	C22—C23—H23	119.3
C12—C10—C9	108.74 (15)	C23—C24—C25	127.04 (19)
C18—C10—C9	113.04 (15)	C23—C24—H24	116.5
C11—C10—C9	108.56 (14)	C25—C24—H24	116.5
C6—C11—C1	114.81 (15)	C26—C25—C30	118.55 (19)
C6—C11—C10	110.32 (15)	C26—C25—C24	119.32 (19)
C1—C11—C10	111.11 (15)	C30—C25—C24	122.08 (18)
C6—C11—H11	106.7	C27—C26—C25	120.8 (2)
C1—C11—H11	106.7	C27—C26—H26	119.6
C10—C11—H11	106.7	C25—C26—H26	119.6
C13—C12—C10	113.86 (16)	C28—C27—C26	119.9 (2)
C13—C12—H12A	108.8	C28—C27—H27	120.1
C10—C12—H12A	108.8	C26—C27—H27	120.1
C13—C12—H12B	108.8	C27—C28—C29	119.5 (2)
C10—C12—H12B	108.8	C27—C28—H28	120.2
H12A—C12—H12B	107.7	C29—C28—H28	120.2
C14—C13—C12	111.37 (16)	C30—C29—C28	121.0 (2)
C14—C13—H13A	109.4	C30—C29—H29	119.5
C12—C13—H13A	109.4	C28—C29—H29	119.5
C14—C13—H13B	109.4	C29—C30—C25	120.2 (2)
C12—C13—H13B	109.4	C29—C30—H30	119.9
H13A—C13—H13B	108.0	C25—C30—H30	119.9
			
C21—O1—C2—C3	−0.3 (2)	C12—C10—C11—C6	179.64 (15)
C21—O1—C2—C1	177.67 (17)	C18—C10—C11—C6	61.13 (18)
C11—C1—C2—C3	−5.0 (3)	C9—C10—C11—C6	−62.16 (18)
C11—C1—C2—O1	177.44 (16)	C12—C10—C11—C1	51.1 (2)
O1—C2—C3—C20	0.0 (2)	C18—C10—C11—C1	−67.37 (19)
C1—C2—C3—C20	−177.72 (19)	C9—C10—C11—C1	169.34 (15)
O1—C2—C3—C5	−176.94 (16)	C18—C10—C12—C13	−71.1 (2)
C1—C2—C3—C5	5.3 (3)	C11—C10—C12—C13	170.62 (16)
C2—C3—C5—C19	101.4 (2)	C9—C10—C12—C13	52.5 (2)
C20—C3—C5—C19	−74.6 (3)	C10—C12—C13—C14	−54.4 (2)
C2—C3—C5—C6	−25.1 (2)	C12—C13—C14—C15	55.7 (2)
C20—C3—C5—C6	158.87 (19)	C13—C14—C15—C17	−173.02 (17)
C3—C5—C6—C7	169.67 (16)	C13—C14—C15—C16	72.4 (2)
C19—C5—C6—C7	46.4 (2)	C13—C14—C15—C9	−54.3 (2)
C3—C5—C6—C11	46.4 (2)	O2—C9—C15—C17	52.1 (2)
C19—C5—C6—C11	−77.0 (2)	C8—C9—C15—C17	−56.2 (2)
C11—C6—C7—C8	−52.9 (2)	C10—C9—C15—C17	171.43 (16)
C5—C6—C7—C8	−179.92 (16)	O2—C9—C15—C16	174.00 (16)
C22—O3—C8—C7	−106.31 (17)	C8—C9—C15—C16	65.7 (2)
C22—O3—C8—C9	129.17 (16)	C10—C9—C15—C16	−66.7 (2)
C6—C7—C8—O3	−77.3 (2)	O2—C9—C15—C14	−65.12 (18)
C6—C7—C8—C9	46.7 (2)	C8—C9—C15—C14	−173.46 (15)
O3—C8—C9—O2	−171.41 (13)	C10—C9—C15—C14	54.2 (2)
C7—C8—C9—O2	66.56 (18)	C2—C3—C20—C21	0.3 (2)
O3—C8—C9—C10	75.14 (18)	C5—C3—C20—C21	176.85 (19)
C7—C8—C9—C10	−46.9 (2)	C3—C20—C21—O1	−0.5 (2)
O3—C8—C9—C15	−58.2 (2)	C2—O1—C21—C20	0.5 (2)
C7—C8—C9—C15	179.73 (16)	C8—O3—C22—O4	0.7 (3)
O2—C9—C10—C12	65.18 (19)	C8—O3—C22—C23	−179.31 (15)
C8—C9—C10—C12	173.04 (15)	O4—C22—C23—C24	−5.4 (3)
C15—C9—C10—C12	−53.6 (2)	O3—C22—C23—C24	174.62 (18)
O2—C9—C10—C18	−173.68 (15)	C22—C23—C24—C25	179.95 (18)
C8—C9—C10—C18	−65.8 (2)	C23—C24—C25—C26	−176.2 (2)
C15—C9—C10—C18	67.5 (2)	C23—C24—C25—C30	1.2 (3)
O2—C9—C10—C11	−53.09 (19)	C30—C25—C26—C27	−1.1 (3)
C8—C9—C10—C11	54.77 (19)	C24—C25—C26—C27	176.37 (18)
C15—C9—C10—C11	−171.88 (16)	C25—C26—C27—C28	−0.6 (3)
C7—C6—C11—C1	−173.43 (15)	C26—C27—C28—C29	1.6 (3)
C5—C6—C11—C1	−49.4 (2)	C27—C28—C29—C30	−0.9 (3)
C7—C6—C11—C10	60.11 (19)	C28—C29—C30—C25	−0.8 (3)
C5—C6—C11—C10	−175.83 (15)	C26—C25—C30—C29	1.8 (3)
C2—C1—C11—C6	26.3 (2)	C24—C25—C30—C29	−175.65 (19)
C2—C1—C11—C10	152.33 (16)		

### Hydrogen-bond geometry (Å, º)

**Table:**

*D*—H···*A*	*D*—H	H···*A*	*D*···*A*	*D*—H···*A*
O2—H2*A*···O1^i^	0.82 (3)	2.28 (3)	3.067 (2)	160 (2)
C18—H18*A*···O3	0.98	2.23	3.039 (2)	139

Symmetry code: (i) *x*−1/2, −*y*−1/2, −*z*+1.
